# Unique Epitopes Recognized by Monoclonal Antibodies against HP-PRRSV: Deep Understanding of Antigenic Structure and Virus-Antibody Interaction

**DOI:** 10.1371/journal.pone.0111633

**Published:** 2014-10-31

**Authors:** Qian Wang, Jinmei Peng, Yan Sun, Jiazeng Chen, Tongqing An, Chaoliang Leng, Lin Li, Hongyuan Zhao, Xin Guo, Xinna Ge, Hanchun Yang, Zhijun Tian

**Affiliations:** 1 Division of Swine Infectious Diseases, National Key Laboratory of Veterinary Biotechnology, Harbin Veterinary Research Institute, the Chinese Academy of Agricultural Sciences, Harbin, China; 2 Key Laboratory of Animal Epidemiology and Zoonosis of Ministry of Agriculture, College of Veterinary Medicine and State Key Laboratory of Agribiotechnology, China Agricultural University, Beijing, China; College of Veterinary Medicine, China

## Abstract

Highly pathogenic porcine reproductive and respiratory syndrome virus (HP-PRRSV) is a member of the genus *Arterivirus* within the family *Arteriviridae*. N and GP3 proteins are the immunodominance regions of the PRRSV viral proteins. To identify the B-cell linear antigenic epitopes within HP-PRRSV N and GP3 proteins, two monoclonal antibodies (mAbs) against N and GP3 proteins were generated and characterized, designated as 3D7 and 1F10 respectively. The mAb 3D7 recognized only HuN4-F112 not the corresponding virulent strain (HuN4-F5). It also recognized two other commercial vaccines (JXA1-R and TJM-F92), but not two other HP-PRRSV strains (HNZJJ-F1 and HLJMZ-F2). The B-cell epitope recognized by the mAb 3D7 was localized to N protein amino acids 7–33. Western blot showed that the only difference amino acid between HuN4-F112-N and HuN4-F5-N did not change the mAb 3D7 recognization to N protein. The epitope targeted by the mAb 1F10 was mapped by truncated proteins. We found a new epitope (68-76aa) can be recognized by the mAb. However, the epitope could not be recognized by the positive sera, suggesting the epitope could not induce antibody in pigs. These results should extend our understanding of the antigenic structure of the N protein and antigen-antibody reactions of the GP3 protein in different species.

## Introduction

Porcine reproductive and respiratory syndrome (PRRS) is an economically devastating viral disease affecting the swine industry worldwide [1]. The disease has many clinical manifestations, but the two most prevalent features are reproductive failure in pregnant sows and respiratory complications in piglets and growing pigs. The etiologic agent, porcine reproductive and respiratory syndrome virus (PRRSV), is an enveloped single-stranded positive-sense RNA virus classified in the order *Nidovirales*, the family *Arteriviridae*, and the genus *Arterivirus*, together with equine arteritis virus (EAV), lactate dehydrogenase-elevating virus (LDV) of mice, and simian hemorrhagic fever virus (SHFV) [2], [3].

The PRRSV genome contains 10 open reading frames (ORF). ORF1a and ORF1b encode viral replicase polyproteins, which are translated immediately upon viral entry and are then proteolytically processed by virally encoded proteinases into 14 mature nonstructural proteins (NSP1α, NSP1β, NSP2-NSP6, NSP7α, NSP7β, and NSP8-NSP12) [4], [5], [6], [7]. The structural-protein-coding region of PRRV encodes eight structural proteins: GP2, E, GP3, GP4, GP5, M, N, and GP5a [8], [9], [10].

In 2006, a highly pathogenic PRRSV (HP-PRRSV) was reported as the main cause of large-scale outbreaks of PRRS with high mortality in China [11], [12], [13]. Epidemics of the atypical disease caused by this emergent HP-PRRSV have resulted in huge economic losses in the Chinese pig industry since 2006 [11], [14]. To control PRRS in China, an attenuated vaccine HuN4-F112 derived from the highly pathogenic HuN4 was developed by passaging HuN4 in Marc-145 cells [15]. This vaccine has been approved for use in China. Previous studies have shown that there are only a few discontinuous amino acid differences between HuN4-F112 and the corresponding virulent strain (HuN4-F5) [16], whereas their biological characteristics differ greatly. It is scientifically important to identify the antigenic differences between these two strains and to determine the molecular mechanisms underlying the attenuation of the virus. The unglycosylated N protein is highly immunogenic in pigs [17]–[19] and in mice [20]–[23]. The GP3 protein is highly glycosylated [17], which plays a role in clearing the viral infection [24] and may be involved in viral neutralization along with the GP5 and M proteins [25]. The localization of viral protein epitopes is very important for determining antigenic structure and virus-antibody interactions at molecular level. In this study, we mapped the B-cell linear epitope of N and GP3 proteins using monoclonal antibodies (mAbs), which helped to open new insights on the structure of the N protein and antibody-antigen reaction in different species of the GP3 protein.

## Materials and Methods

### Ethics statement

Animal experiments were approved by Animal Ethics Committee of Harbin Veterinary Research Institute of the Chinese Academy of Agricultural Sciences (CAAS) and performed in accordance with animal ethics guidelines and approved protocols. The Animal Ethics Committee approval number was SYXK (Hei) 2011022.

### Viruses, cells, plasmids, and sera

HP-PRRSV HuN4 (GenBank accession: EF635006) was isolated in China and its pathogenicity has been characterized [13], [15], [26]. The vaccine strain, HuN4-F112, was obtained by culturing the parent strain, HP-PRRSV HuN4, with passage in Marc-145 cells for 112 passages [15]. TJM-F92 and JXA1-R are commercial vaccines available in China. HP-PRRSV HNZJJ-F1 and HLJMZ-F2 were passaged and the passages are retained in our laboratory. The Marc-145 cell line, SP2/0 cell line, and HEK293T cell line were purchased from ATCC. The eukaryotic expression vectors pCAGGS-HuN4-F112-GP2, GP3, GP4, GP5, M, and N are retained in our laboratory. BL21-pGEX-6P-1-HuN4-GP3-(1-171aa, 41-100aa, 41-55aa, 56-70aa, and 63-77aa) were conserved by our laboratory. PRRSV positive sera (4^#^, 7^#^, 28^#^, and 71^#^) were obtained from piglets that was immunized with HP-PRRSV HuN4 vaccine strain (HuN4-F112) or/and with HP-PRRSV virulent strain (HuN4-F5) [27].

### Production and characterization of monoclonal antibodies (mAbs) against HuN4-F112

Female BALB/c mice aged 4–6 weeks (from the Laboratory Animal Center of Harbin Veterinary Research Institute, CAAS) were immunized intraperitoneally three times at two-weekly intervals with HuN4-F112 (10^7.0^ TCID_50_), in Freund’s complete adjuvant (Sigma, St. Louis, MO, USA) for the first immunization and in Freund’s incomplete adjuvant (FICA) for the other two immunizations, and then boosted intraperitoneally with virus only. Three days after the final booster injection, mice were euthanized by Carbon Dioxide and then spleen cells were fused with SP2/0 cells using 50% (v/v) polyethylene glycol (Sigma, St. Louis, MO, USA). The fused cells were cultured successively in Dulbecco’s Modified Eagle’s Medium (DMEM) (Gibco BRL Co. Ltd., USA) containing HAT (Sigma, St. Louis, MO, USA), HT (Sigma, St. Louis, MO, USA) and then in DMEM supplemented only with 20% fetal bovine serum (Hyclone Laboratories Inc., South Logan, UT, USA). The isotypes of the mAbs produced were determined with the Pierce Rapid ELISA Mouse mAb Isotyping Kit (Thermo Scientific, Massachusetts, USA), according to the manufacturer’s instructions.

### Indirect immunofluorescence assay (IFA)

The hybridomas were screened with IFA for their secretion of the desired antibodies. In brief, Marc-145 cell monolayers were infected with HuN4-F112 at a multiplicity of infection (MOI) of 0.1 and incubated for 24–48 h at 37°C. The cells were harvested by digestion and centrifugation, and washed once with phosphate-buffered saline (PBS). Eight-hole glass slides were coated with the infected cells, air dried, and fixed with cold acetone. IFA was performed using the hybridoma supernatants as the primary antibody and fluorescein-isothiocyanate (FITC)-conjugated goat anti-mouse IgG (Zsbio, Beijing, China) as the secondary antibody. The samples were analyzed with a fluorescence microscope (Nikon TS100, Japan). The selected clones were subcloned by limiting dilution. Ascites fluids were produced in FICA-primed BALB/c mice.

Transient transfection was performed to identify the structural proteins that combined with the mAbs. 293T cells were transiently transfected with the eukaryotic expression vector pCAGGS-HuN4-F112-GP2, GP3, GP4, GP5, M and N using X-tremeGENE HP DNA Transfection Reagent (Roche, Basel, Switzerland), according to the manufacturer’s protocol. pCAGGS-transfected cells were used as the negative control. And Marc-145 cell monolayers were infected with HuN4-F5, TJM-F92, JXA1-R, HNZJJ-F1 and HLJMZ-F2 at a multiplicity of infection (MOI) of 0.1. The cells were harvested and IFA was performed as described above.

### Sequencing and alignment of the PRRSV ORF7 gene

Viral RNAs (the six strains mentioned above) were extracted with the RNeasy Plus Mini Kit (Qiagen, Dusseldorf, Germany), and RT-PCR was performed as described previously [28], using forward and reverse primers containing *Eco*R I and *Xho* I recognition sites, respectively, to amplify the complete ORF7 sequence (Table 1). The PCR product was cloned into the pMD18-T vector (Takara, Dalian, Japan) and confirmed by sequencing. Sequence alignments were performed with DNAMAN (Lynnon Biosoft, USA).

**Table 1:** Primer sets for the amplification of the ORF7 gene and its truncated fragments.

Name of fragment	Sequences of PCR primers (5′-3′)	Position of nucleotide acid in ORF7	Position of amino acid in N protein
F112-N	F: GGAATTC *GCCACC*ATGCCAAATAACAAC	1–372	1–123
	R: CCTCGAG **TCA**TGCTGAGGGTGATGCTGT		
NF1	F: CGGAATTCATGCCAAATAACAACGGC	1–126	1–42
	R: CTCGAG **TTA**CCCCGGTCCCTTGCC		
NF2	F: GAATTC AAGATCATCGCCCAAC	82–207	28–69
	R: CTCGAG **TTA**AGGGGTAAAGTGATG		
NF3	F: GAATTC TTCCCTCTAGCGACTGAAG	163–288	55–96
	R: CTCGAG **TTA**CCCTGAATCTGACAGG		
NF4	F: GAATTC GCATTCAATCAGGGCGCT	244–372	82–123
	R: CTCGAG **TCA**TGCTGAGGGTGATGCT		
NF1-1	R: CTCGAG **TTA**CTTGCCTCTGGACTGGT	1–117	1–39
NF1-2	R: CTCGAG **TTA**GGACTGGTTTTGTTGGGC	1–108	1–36
NF1-3	R: CTCGAG **TTA**TTGTTGGGCGATGATCTT	1–99	1–33
NF1-4	R: CTCGAG **TTA**GATGATCTTACCCAGC	1–90	1–30
NF1-5	R: CTCGAG **TTA**ACCCAGCATTTGGCAC	1–81	1–27
NF1-3-1	F: GAATTC AACAACGGCAAGCAGCAAAAG	10–99	4–33
NF1-3-2	F: GAATTC AAGCAGCAAAAGAAAAAGAAGGG	19–99	7–33

In these primer sets, the forward and reverse primers contain *Eco*RI and *Xho*I recognition sites, respectively (underlined). Bold font indicates termination codons. Italic font indicates the Kozak sequence, and this forward primer was used to construct the eukaryotic expression vector pCAGGS-F112-N. NF1 shared the same forward primer with NF1-1 to NF1-5. In the same way, NF1-3 shared the same reverse primer with NF1-3-1 and NF1-3-2.

### Expression of recombinant HuN4-F5-N, HuN4-F112-N

The complete ORF7 genes, digested from pMD18-T-HuN4-F5-N and pMD18-T-HuN4-F112-N, were ligated into the expression vector pGEX-6p-1 (Invitrogen, Scotland, UK). *Escherichia coli* BL21 cells (Takara) were transformed with the confirmed recombinant plasmids and the respective proteins were expressed. Sodium dodecyl sulfate polyacrylamide gel electrophoresis (SDS-PAGE) was performed to analyze the glutathione S-transferase (GST)-HuN4-F5-N and GST-HuN4-F112-N fusion proteins. HuN4-F5 and HuN4-F112 were isolated ultracentrifugally from cell pellets harvested by centrifugation and their immune activity was analyzed directly with Western blot.

### Western blot

The cell-expressed GST and the GST-N fusion proteins were separated by 12% SDS-PAGE and the protein samples were then transferred onto nitrocellulose filter membrane (PALL, New York, USA). The membranes were incubated with ascites fluid and anti-GST mAb as the primary antibody. After the membranes were rinsed with PBS, each membrane was treated with IRDye-700-conjugated goat anti-mouse IgG (LI-COR Biosciences, USA) as the secondary antibody. The proteins were visualized by scanning the membranes with the LI-COR Odyssey infrared image system (LI-COR Biotechnology, USA).

### Localization of B-cell linear epitope using overlapping F112-N protein fragments

The ORF7 gene was each divided into four overlapping fragments: NF1-NF4. Specific primers (Table 1) were used to amplify these fragments. The PCR products amplified from these four fragments were cloned separately into the pGEX-6p-1 expression vector and used to transform *E. coli* BL21 (DE3) cells (Tiangen, Beijing, China), in which the corresponding proteins were expressed. The recombinant proteins were identified by SDS-PAGE and Western blot, as described above. Based on an epitope analysis of the larger fragment of the N protein, NF1 (amino acids 1–42) was serially truncated three by three from the N- and C-termini, respectively (Table 1). These fragments were identified with SDS-PAGE and Western blot, as described above.

### Identification of B-cell linear epitope in the GP3 protein

BL21-pGEX-6P-1-HuN4-GP3-(1-171aa, 41-100aa, 41-55aa, 56-70aa, and 63-77aa) were induced and expressed. The protein samples were identified with SDS-PAGE and Western blot using the mAb 1F10, as described above.

Based on an epitope analysis of the larger fragments of the GP3 protein, GP3 63-77aa was serially truncated one by one from the N- and C- termini, respectively (Table 2). The complementary oligonucleotide pairs encoding each peptide were synthesized, annealed and cloned them into the *BamH*I and *Xho*I sites of pGEX-6p-1 expression vector, resulting in sixteen recombinant plasmids. The GST fusion proteins were expressed and used to screen the mAb by Western blot as described above.

**Table 2:** Primer sets for the amplification of the ORF3 gene and its truncated fragments.

Name of fragment	Sequences of PCR primers (5′–3′)
F112-GP3	F: GCGCGCGGATCCATGGCTAATAGCTGTAC
	R: CTCGAG **CTA**TCGCCGTGCGGCACTGA
GP3 63-76	F: GATCC GCCGCTGAGATCCTTGAGCCCGGCAAGTCTTTTTGGTGCAGG**TAA** C
	R: TCGAG **TTA**CCTGCACCAAAAAGACTTGCCGGGCTCAAGGATCTCAGCGGCG
GP3 63-75	F: GATCC GCCGCTGAGATCCTTGAGCCCGGCAAGTCTTTTTGGTGC**TAA** C
	R: TCGAG **TTA**GCACCAAAAAGACTTGCCGGGCTCAAGGATCTCAGCGGCG
GP3 63-74	F: GATCC GCCGCTGAGATCCTTGAGCCCGGCAAGTCTTTTTGG**TAA** C
	R: TCGAG **TTA**CCAAAAAGACTTGCCGGGCTCAAGGATCTCAGCGGCG
GP3 63-73	F: GATCC GCCGCTGAGATCCTTGAGCCCGGCAAGTCTTTT**TAA** C
	R: TCGAG **TTA**AAAAGACTTGCCGGGCTCAAGGATCTCAGCGGCG
GP3 63-72	F: GATCC GCCGCTGAGATCCTTGAGCCCGGCAAGTCT**TAA** C
	R: TCGAG **TTA**AGACTTGCCGGGCTCAAGGATCTCAGCGGCG
GP3 63-71	F: GATCC GCCGCTGAGATCCTTGAGCCCGGCAAG**TAA** C
	R: TCGAG **TTA**CTTGCCGGGCTCAAGGATCTCAGCGGCG
GP3 64-77	F: GATCC GCTGAGATCCTTGAGCCCGGCAAGTCTTTTTGGTGCAGGATA**TAA** C
	R: TCGAG **TTA**TATCCTGCACCAAAAAGACTTGCCGGGCTCAAGGATCTCAGCG
GP3 65-77	F: GATCC GAGATCCTTGAGCCCGGCAAGTCTTTTTGGTGCAGGATA**TAA** C
	R: TCGAG **TTA**TATCCTGCACCAAAAAGACTTGCCGGGCTCAAGGATCTCG
GP3 66-77	F: GATCC ATCCTTGAGCCCGGCAAGTCTTTTTGGTGCAGGATA**TAA** C
	R: TCGAG **TTA**TATCCTGCACCAAAAAGACTTGCCGGGCTCAAGGATG
GP3 67-77	F: GATCC CTTGAGCCCGGCAAGTCTTTTTGGTGCAGGATA**TAA** C
	R: TCGAG **TTA**TATCCTGCACCAAAAAGACTTGCCGGGCTCAAGG
GP3 68-77	F: GATCC GAGCCCGGCAAGTCTTTTTGGTGCAGGATA**TAA** C
	R: TCGAG **TTA**TATCCTGCACCAAAAAGACTTGCCGGGCTCG
GP3 69-77	F: GATCC CCCGGCAAGTCTTTTTGGTGCAGGATA**TAA** C
	R: TCGAG **TTA**TATCCTGCACCAAAAAGACTTGCCGGGG
GP3 70-77	F: GATCC GGCAAGTCTTTTTGGTGCAGGATA**TAA** C
	R: TCGAG **TTA**TATCCTGCACCAAAAAGACTTGCCG
GP3 71-77	F: GATCC AAGTCTTTTTGGTGCAGGATA**TAA** C
	R: TCGAG **TTA**TATCCTGCACCAAAAAGACTTG
GP3 72-77	F: GATCC TCTTTTTGGTGCAGGATA**TAA** C
	R: TCGAG **TTA**TATCCTGCACCAAAAAGAG
GP3 73-77	F: GATCC TTTTGGTGCAGGATA**TAA** C
	R: TCGAG **TTA**TATCCTGCACCAAAAG
GP3 69-76	F: GATCC CCCGGCAAGTCTTTTTGGTGCAGG**TAA** C
	R: TCGAG **TTA**CCTGCACCAAAAAGACTTGCCGGGG
GP3 68-76	F: GATCC GAGCCCGGCAAGTCTTTTTGGTGCAGG**TAA** C
	R: TCGAG **TTA**CCTGCACCAAAAAGACTTGCCGGGCTCG

In the F112-GP3 primers, the forward and reverse primers contain *Bam*HI and *Xho*I recognition sites, respectively (underlined). Other oligomeric nucleic acid fragments were annealed directively and be used for amino acids pursue absence, which also contain *Bam*HI and *Xho*I recognition sites (underlined). Bold font indicates termination codons.

### Sequence alignmenst of the epitope recognized by mAb 3D7 and 1F10

The nucleotide sequences of PRRSV strains from different countries were retrieved from GenBank. The amino acid sequences of the identified B-cell epitope were aligned using the DNAstar MegAlign software (DNASTAR Inc., Madison, WI, USA). The representative PRRSV strains are listed in Table 3.

**Table 3:** PRRSV strains cited in this study.

Isolate	Accession number	Type	Virulence
HUN4	EF635006	Type II	High Virulence
HUB1	EF075945	Type II	High Virulence
JX143	EU708726	Type II	High Virulence
JXA1	EF112445	Type II	High Virulence
JXwn06	EF641008	Type II	High Virulence
Jiangxi-3	EU200961	Type II	High Virulence
SX2009	FJ895329	Type II	High Virulence
SY0608	EU144079	Type II	High Virulence
WUH1	EU187484	Type II	High Virulence
YN2008	EU880435	Type II	High Virulence
CH-1a	AY032626	Type II	Virulence
CH2002	EU880438	Type II	Virulence
BJ-4	AF331831	Type II	Virulence
HN1	AY457635	Type II	Virulence
P129	AY585241	Type II	Virulence
VR2332	AY150564	Type II	Virulence
JXA1 P80	FJ548853	Type II	Vaccine
CH-1R	EU807840	Type II	Vaccine
MLV	AF066183	Type II	Vaccine
Lelystad virus	M96262	Type I	Virulence
NMEU09-1	GU047345	Type I	Virulence

### The mAb 3D7 responses during different passages of HP-PRRSV

HP-PRRSV, HLJMZ-F2, in addition to HuN4, was passaged in Marc-145 cells about 10 times. The different passages were used to inoculate Marc-145 cell monolayers grown for 36 h before viral inoculation. IFA was performed 48 h after inoculation using the the mAb, as described above.

### GP3 epitope’s reactivity with PRRSV-positive sera

To detect whether PRRSV-positive sera contained antibodies against the GP3 epitope identified above, the epitope was analyzed by Western blot using PRRSV-positive sera.

## Results

### Development and identification of mAbs

**Figure 1:**
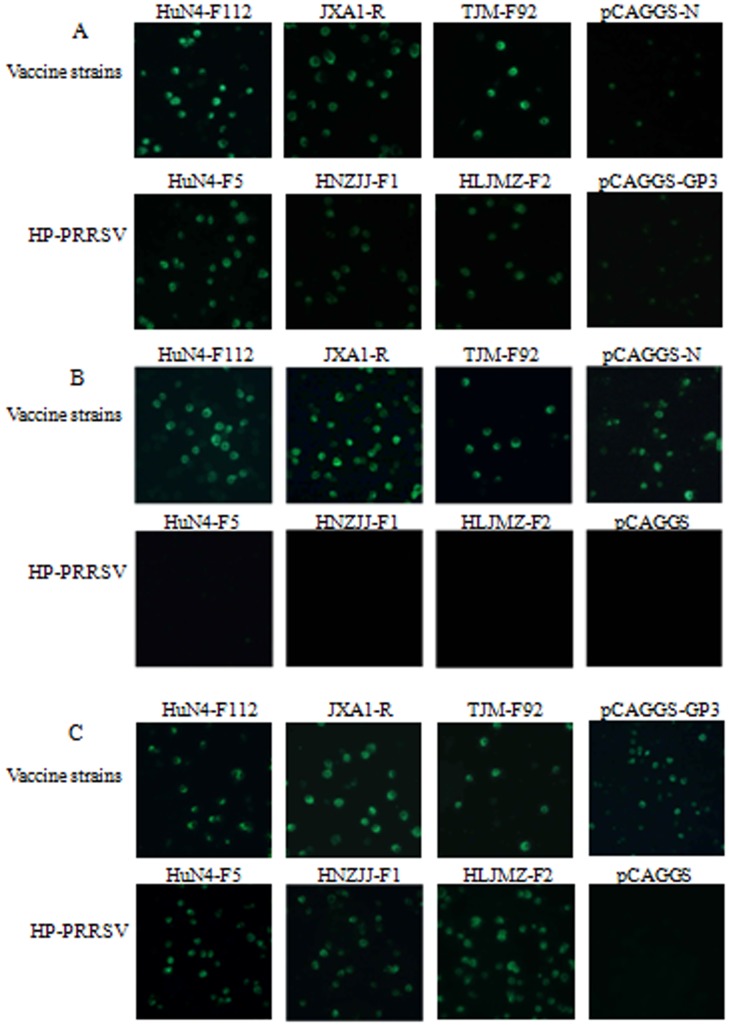
Reactivity of mAbs 3D7 (B) and 1F10 (C) with HP-PRRSV HuN4 and vaccine strains in Marc-145 cells and transiently transfected 293T cells expressing expressing N and GP3 proteins. HuN4-F112 (attenuated in our laboratory), JXA1-R, and TJM-F92 are commercial vaccines used in China. HuN4, HNZJJ, and HLJMZ are HP-PRRSVs isolated by our laboratory. The two mAbs 3D7 and 1F10 recognized N and GP3 protein expressed by transiently transfected cells. 293T cells pCAGGS-transfected were used as a negative control. Marc-145 cells infected by PRRSV staining with the mAb 3F7 and 293T cells pCAGGS-N/GP3-transfected staining with the mAbs 2E9 and 4G5 were used as positive controls (A). Magnification 200×.

Two mAbs, 3D7 and 1F10, were generated with IFA detection using HuN4-F112 as the antigen source. The two mAbs specifically recognized N and GP3 proteins expressed by transiently transfected cells (Fig. 1). The mAb 3D7 reacted positively with the Marc-145 cells infected with the HP-PRRSV vaccine strains (JXA1-R and TJM-F92). However, they did not recognize the HP-PRRSVs HuN4-F5, HNZJJ-F1, and HLJMZ-F2 (Fig. 1). The mAb 1F10 recognized the HP-PRRSV HuN4, and commercial vaccine strains (JXA1-R and TJM-F92) (Fig. 1). The isotypes of the mAbs 3D7 and 1F10 were identified with the Pierce Rapid ELISA Mouse mAb Isotyping Kit as IgG1. A neutralization assay was performed as described previously [29], and indicated that the two mAbs did not neutralize PRRSV (data not shown).

### Mutation analysis of N protein by the mAb 3D7

**Figure 2:**
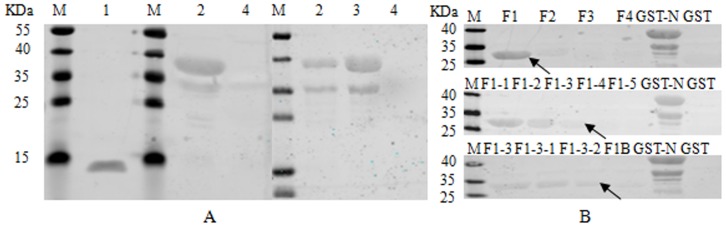
Identification of the mAb 3D7 epitope by Western blot. Western blot analysis of GST-HuN4-F112/F5-N fusion proteins with the mAb 3D7 (A). Lane 1: ultracentrifugal HuN4-F112; lane 2: GST-HuN4-F112-N; lane 3: GST-HuN4-F5-N; lane 4: GST. Truncated fragments were detected with the mAb 3D7 (B). The mAb 3D7 specifically reacted with N protein fragment NF1-3-2 (amino acids 7–33) after three rounds of truncation. F1B is the fragment (amino acids 10–33) identified previously as not recognized by the mAb 3D7 (data not shown), used here as a negative control.

The IFA result showed that the mAb 3D7 recognized HuN4-F112, but not HuN4-F5 (Fig. 1). When the ORF7 genes of the six strains mentioned above were sequenced, the sequences of the HP-PRRSV strains had no identical difference point compared with those of the vaccine strains. When the N proteins of HuN4-F112 and HuN4-F5, which only differed at one amino acid position (K^11^ and R^11^, respectively) were expressed in *E. coli* BL21, both reacted with the mAb 3D7 (Fig. 2A). Therefore, the amino acid at residue 11 is not a key amino acid.

### Identification of B-cell epitopes recognized by the mAbs 3D7 and 1F10

The results described above confirmed that the mAb 3D7 recognized the N protein both in Western blot and in IFA. Four overlapping fragments (NF1, NF2, NF3, and NF4) from ORF7 gene were prepared by PCR and cloned into an expression vector, pGEX-6p-1, for expression as GST fusion proteins. After nucleotide sequencing, the recombinant protein fragments encoded by these constructs were expressed in *E. coli* BL21 (DE3) cells and identified with SDS-PAGE and Western blot with the mAb 3D7. The results showed that NF1 (amino acids 1–42) reacted with the mAb 3D7. NF1 (amino acids 1–42) was then serially truncated three by three from the N- and C-termini, respectively. The NF1-3-2 (amino acids 7–33) was recognized by the mAb 3D7 (Fig. 2B).

**Figure 3:**
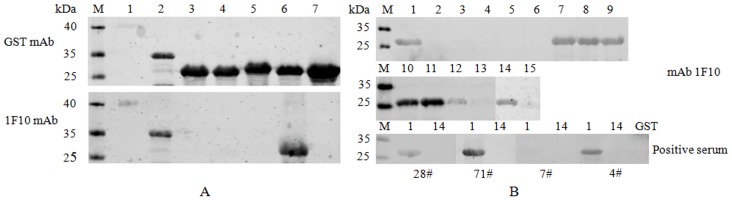
The truncated GP3 fragments (A) and the pursue absence GP3-63-77aa fragments (B) were identified by Western blot with the anti-GST mAb, the mAb 1F10, and positive sera. (A) M: protein marker; 1: GST-GP3-1-171aa; 2: GST-GP3-41-100aa; 3: GST-GP3-41-55aa; 4: GST-GP3-56-70aa; 5: GST-GP3-48-62aa; 6: GST-GP3-63-77aa; 7: GST. (B) M: protein marker; 1: GST-GP3-63-76aa; 2: GST-GP3-63-75aa; 3: GST-GP3-63-74aa; 4: GST-GP3-63-73aa; 5: GST-GP3-63-72aa; 6: GST-GP3-63-71aa; 7: GST-GP3-64-77aa; 8: GST-GP3-65-77aa; 9: GST-GP3-66-77aa; 10: GST-GP3-67-77aa; 11: GST-GP3-68-77aa; 12: GST-GP3-69-77aa; 13: GST-GP3-70-77aa; 14: GST-GP3-68-76aa; 15: GST-GP3-69-76aa. The deduced epitope (69-76aa) was vertified not to be a true one, while the motif (68-76aa) was the epitope recognized by the mAb 1F10. But the epitope was not recognized by positive sera. The positive sera 4^#^ and 7^#^ were pig hyperimmune sera with high titer of neutralizing antibodies against HP-PRRSV, which were obtained from pigs immunized by HuN4-F112 once and then inoculated with HP-PRRSV HuN4 virulent strain (HuN4-F5) three times. The positive serum 28^#^ was collected from a pig inoculated with HP-PRRSV HuN4 virulent strain (HuN4-F5) at 14DPI. A pig was immunized with HuN4-F112 and then inoculated with HP-PRRSV HuN4 virulent strain (HuN4-F5) at 21DPI, the positive serum 71^#^ was collected from the pig after 3 weeks.

Because the mAb 1F10 recognized both HuN4-F112 and HuN4 (Fig. 1), existing BL21-pGEX-6P-1-HuN4-GP3-(1-171aa, 41-100aa, 41-55aa, 56-70aa, and 63-77aa) were induced for 4 h for expression. The recombinant proteins were identified by Western blot using the mAb 1F10. As shown in Fig. 2, the mAb 1F10 recognized GP3 63-77aa. To further clarify the position of the linear antigenic epitope within this domain, short peptides truncated one by one were expressed and identified by Western blot. The data showed that the mAb 1F10 may recognize the minimal epitope GP3 69-76aa. In order to further verify the result, the fusion polypeptides GP3 69-76aa and GP3 68-76aa were expressed and identified by Western blot. The result indicated that the core sequence recognized by the mAb 1F10 was ^68^PGKSFWCR^76^ (Fig. 3).

### Sequence alignments of the two epitopes of different PRRSV strains

**Figure 4:**
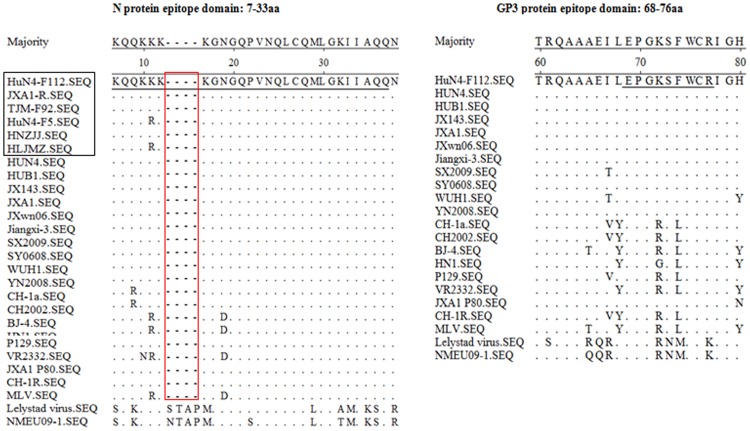
Multiple sequence alignments of the epitopes of the N and GP3 proteins of HP-PRRSV, classical PRRSV isolates, and vaccine strains. The amino acid sequences of the epitopes identified are underlined. The strains in square frame are sequenced in our laboratory. Strigulas (red square frame) stands in for the amino acids deleted from the North American PRRSV relative to the sequence of European virus. The amino acid sequences are aligned using the DNAstar MegAlign software.

An alignment of the amino acid sequences of the different PRRSV isolates revealed that the epitope on protein N (amino acids 7–33) was relatively conserved among the HP-PRRSV strains, but differed at one amino acid between HuN4-F112 (K^11^) and HuN4-F5 (R^11^) (Fig. 2). We then expressed the mutated (R^11^) N protein, which did not alter the response between the mutated N protein and the mAb 3D7 (data described in 3.2). The result also suggest that the failure of the mAb 3D7 to combine with the HP-PRRSV N protein was unrelated to its amino acid differences. Another alignment revealed that the epitope on protein GP3 (68-76aa) was highly conserved among the HP-PRRSV strains, but have two amino acid differences within the epitope among the classical strains. However, there was four amino acids varied between the HP-PRRSV strains and European-type strains (Fig. 4).

### Identification of the different passages of HP-PRRSV by the mAb 3D7

**Figure 5:**
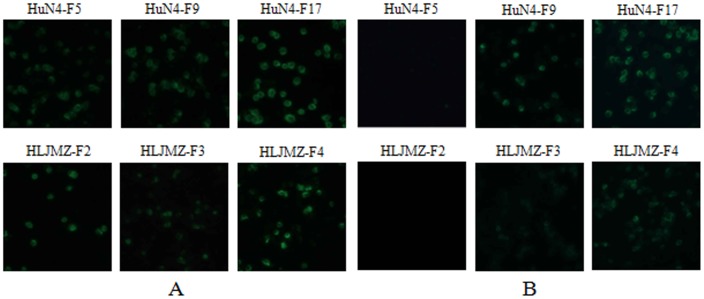
Reactivity of the mAb 3D7 (B) with two HP-PRRSV strains at different passages. The mAb 3D7 reacted with HuN4-F9/F17 and HLJMZ-F3/F4 passages. The mAb 3F7 (A) was used as a positive control.

HP-PRRSV strains HLJMZ-F2 and HuN4-F5 were passaged about 10 times in Marc-145 cells. IFA was used to determine at which passage of the two HP-PRRSV strains their N protein were recognized by the mAb 3D7. mAb 3D7 did not recognize HuN4-F5, but did recognize HuN4-F9 and HuN4-F17 (Fig. 5). Interestingly, these three passages did not contain the amino acid mutation observed in the N protein [16]. Therefore, although the mAb combined with the same epitope, a different antibody response was induced. The other HP-PRRSV strain, HLJMZ-F2, was recognized by the mAb even after a single passage (Fig. 5). Under conditions similar to those used to assay HuN4, the ORF7 genes were not sequenced, but we assumed that the passages of HLJMZ also contained no amino acid mutation in the N protein. Therefore, we concluded that the antigenicity of HP-PRRSV N protein passaged in Marc-145 cells changed rapidly, but in a way that was unrelated to the amino acid sequences of its N protein.

### GP3 epitope’s reactivity with PRRSV-positive serum

To determine whether pigs infected with PRRSV produced antibodies against the epitope (^68^PGKSFWCR^76^), the fusion polypeptide identified above was analyzed by Western blot using PRRSV positive sera of HP-PRRSV. Western blot analysis demonstrated that the anti-sera did not recognize the epitope, indicating that the epitope was not an antigenic epitope in the HP-PRRSV-infected pigs.

## Discussion

In May 2006, a highly pathogenic PRRS, characterized by high fever and a high proportion of deaths in pigs of all ages, emerged in some swine farms in Jiangxi Province. After the initial outbreak, the disease spread rapidly to most provinces in China, resulting more than one million deaths [11], [13]. Because the effectiveness of the live attenuated North-American-type PRRSV vaccines currently in use was limited against HP-PRRSV infections, a live attenuated vaccine derived from the HP-PRRSV HuN4 strain was developed and approved in China. Further study showed that a few discontinuous amino acid mutations existed between HuN4-F112 and its virulent strain (HuN4-F5) [16], whereas its biological characteristics had changed greatly, especially its virulence. Are these amino acid mutations sufficient to alter the virus from a highly pathogenic strain to a nonvirulent strain? We inferred that changes in the antigenicity of the virus must occur during its passage in Marc-145 cells. mAbs are an important tool for identifying the antigenicity of PRRSV and were also used to identify the antigenic differences between HuN4-F112 and HuN4-F5 in this study.

In the present study, two mAbs, 3D7 and 1F10, against the protein of the HP-PRRS vaccine, were prepared from the native virus immunized mice. BALB/c mice were immunized with the virus to stimulate antibody production in vivo, to produce the mAbs more similar to porcine serum antibodies. These mAbs were readily blocked by positive serum against PRRSV. The mAb 3D7 recognized the HP-PRRS vaccines, but not HP-PRRSV strains. Epitope mapping indicated that mAb 3D7 recognized the long epitope (amino acids 7–33). The N protein was truncated many times, resulting in several fragments of 10–24 amino acids, but no fragment was recognized by the mAb 3D7 (data not shown). Therefore, we inferred that the epitope may have been truncated, or that the epitope may be discontinuous. Sequence alignment suggested that the N protein epitope is conserved among the HP-PRRSV and vaccine strains, except at a single amino acid position at residue 11. A previous study identified five domains with antigenic importance in a reference North-American-type strain, localized at amino acids 30–52, 37–52, 52–69, 69–112, and 112–123 [20], [21]. The N-protein epitope (amino acids 7–33) was first identified in this study, confirming that the N protein is highly immunogenic. In the meantime, we found that when HP-PRRSV isolates HuN4 and HLJMZ were passaged in Marc-145 cells, the mAb 3D7 reacted with the higher passages virus not lower passages virus. Maybe the viral protein had changed, but not in its amino acid sequence. These changes may involve protein modifications, changes in the interaction between the viral protein and host proteins, or even between different viral proteins. The cause of this different response to the mAb requires further study. These results demonstrate that the mAb can be used to distinguish HP-PRRSV strains from attenuated PRRSV strains. GP3 is highly antigenic [30]–[32]. Using a phage display library, two epitopes spanning aa regions 60–85 and 243–250 of a EU GP3 protein were identified [33]. Two other GP3 epitopes of an NA strain were found and located at aa 74–85 and 67–74 [34]. In the current study, we have expressed a series of peptides from HuN4-F112 GP3 protein and identified by Western blot. According to the results, deduced motif of the epitope (GP3 69-76aa) was expressed. Our data confirmed that GP3 69-76aa is not the minimal unit of this epitope, while GP3 68-76aa is actually the epitope because of the reactivity with the mAb 1F10. However the epitope was not recognized by PRRSV-positive sera of swine. Maybe mAbs screened epitopes cannot truly reflect the antigen and the antibody reaction during viral infection [35].

In summary, one mAb directed against the PRRSV N protein was developed, which recognized one long epitope (amino acids 7–33) and can also distinguish HP-PRRSV from commercial vaccines. A sequence analysis revealed that the failure of the mAb to recognize the virulent strain (HuN4-F5) was unrelated to the amino acid sequences of its epitope. And we also identified a conserved B-cell epitope precisely on PRRSV GP3 protein. These results should extend our understanding of the antigenic structure of the N protein and antigen-antibody reaction in different species of the GP3 protein.

## References

[1] Neumann EJ, Kliebenstein JB, Johnson CD, Mabry JW, Bush EJ, et al (2005) Assessment of the economic impact of porcine reproductive and respiratory syndrome on swine production in the United States. J Am Vet Med Assoc 227: 385–392. 16121604 10.2460/javma.2005.227.385

[2] Benfield DA, Nelson E, Collins JE, Harris L, Goyal SM, et al (1992) Characterization of swine infertility and respiratory syndrome (SIRS) virus (isolate ATCC VR-2332). J Vet Diagn Invest 4: 127–133. 1616976 10.1177/104063879200400202

[3] Cavanagh D (1997) Nidovirales: a new order comprising Coronaviridae and Arteriviridae. Arch Virol 142: 629–633. 9349308

[4] den Boon JA, Faaberg KS, Meulenberg JJ, Wassenaar AL, Plagemann PG, et al (1995) Processing and evolution of the N-terminal region of the arterivirus replicase ORF1a protein: identification of two papainlike cysteine proteases. J Virol 69: 4500–4505. 7769711 10.1128/jvi.69.7.4500-4505.1995 PMC189193

[5] Snijder EJ, Meulenberg JJ (1998) The molecular biology of arteriviruses. J Gen Virol 79: 961–979. 9603311 10.1099/0022-1317-79-5-961

[6] van Aken D, Zevenhoven-Dobbe J, Gorbalenya AE, Snijder EJ (2006) Proteolytic maturation of replicase polyprotein pp1a by the nsp4 main proteinase is essential for equine arteritis virus replication and includes internal cleavage of nsp7. J Gen Virol 87: 3473–3482. 17098961 10.1099/vir.0.82269-0

[7] Fang Y, Snijder EJ (2010) The PRRSV replicase: exploring the multifunctionality of an intriguing set of nonstructural proteins. Virus Res 154: 61–76. 20696193 10.1016/j.virusres.2010.07.030 PMC7114499

[8] Meulenberg JJ, Petersen-den Besten A, De Kluyver EP, Moormann RJ, Schaaper WM, et al (1995) Characterization of proteins encoded by ORFs 2 to 7 of Lelystad virus. Virology 206: 155–163. 7831770 10.1016/S0042-6822(95)80030-1 PMC7130653

[9] Bautista EM, Meulenberg JJ, Choi CS, Molitor TW (1996) Structural polypeptides of the American (VR-2332) strain of porcine reproductive and respiratory syndrome virus. Arch Virol 14: 1357–1365. 10.1007/BF01718837 8774694

[10] Johnson CR, Griggs TF, Gnanandarajah J, Murtaugh MP (2011) Novel structural protein in porcine reproductive and respiratory syndrome virus encoded by an alternative ORF5 present in all arteriviruse. J Gen Virol 92: 1107–1116. 21307222 10.1099/vir.0.030213-0 PMC3139420

[11] Tian K, Yu X, Zhao T, Feng Y, Cao Z, et al (2007) Emergence of fatal PRRSV variants: unparalleled outbreaks of atypical PRRS in China and molecular dissection of the unique hallmark. PLoS one 2: e526. 17565379 10.1371/journal.pone.0000526 PMC1885284

[12] Li Y, Wang X, Bo K, Wang X, Tang B, et al (2007) Emergence of a highly pathogenic porcine reproductive and respiratory syndrome virus in the Mid-Eastern region of China. Vet J 174: 577–584. 17869553 10.1016/j.tvjl.2007.07.032

[13] Tong GZ, Zhou YJ, Hao XF, Tian ZJ, An TQ, et al (2007) Highly pathogenic porcine reproductive and respiratory syndrome, China. Emerg Infect Dis 13: 1434–1436. 18252136 10.3201/eid1309.070399 PMC2857295

[14] Yang HC (2007) Prevalence status of “swine high fever syndrome” (atypical PRRS). Swine Ind Sci 1: 77–80 (in Chinese)..

[15] Tian ZJ, An TQ, Zhou YJ, Peng JM, Hu SP, et al (2009) An attenuated live vaccine based on highly pathogenic porcine reproductive and respiratory syndrome virus (HP-PRRSV) protects piglets against HP-PRRS. Vet Microbiol. 138: 34–40. 10.1016/j.vetmic.2009.03.003 19339125

[16] Zhou YJ, Tian ZJ, Hao XF, An TQ, Yu H, et al (2011) Molecular characterization of highly pathogenic porcine reproductive and respiratory syndrome virus (HuN4) and its attenuation strain. Chin J Anim Infect Dis 19: 1–10 (in Chinese)..

[17] Meulenberg JJ, Petersen-den Besten A, De Kluyver EP, Moormann RJ, Schaaper WM, et al (1995a) Characterization of proteins encoded by ORFs 2 to 7 of Lelystad virus. Virology 206: 155–163. 7831770 10.1016/S0042-6822(95)80030-1 PMC7130653

[18] Meulenberg JJ, Petersen-den Besten A, de Kluyver EP, Moormann RJ, Schaaper WM, et al (1995b) Characterization of structural proteins of Lelystad virus. Adv Exp Med Bio 380: 271–276. 8830491 10.1007/978-1-4615-1899-0_43

[19] Loemba HD, Mounir S, Mardassi H, Archambault D, Dea S (1996) Kinetics of humoral immune response to the major structural proteins of the porcine reproductive and respiratory syndrome virus. Arch Virol 141: 751–761. 8645111 10.1007/BF01718333 PMC7086943

[20] Nelson EA, Christopher-Hennings J, Drew T, Wensvoort G, Collins JE, et al (1993) Differentiation of U.S. and European isolates of porcine reproductive and respiratory syndrome virus by monoclonal antibodies. J Clin Microbiol 31: 3184–3189. 7508455 10.1128/jcm.31.12.3184-3189.1993 PMC266373

[21] Drew TW, Meulenberg JJ, Sands JJ, Paton DJ (1995) Production, characterization and reactivity of monoclonal antibodies to porcine reproductive and respiratory syndrome virus. J GenVirol 76: 1361–1369. 10.1099/0022-1317-76-6-1361 7782765

[22] van Nieuwstadt AP, Meulenberg JJ, van Essen-Zanbergen A, Petersen-den Besten A, Bende RJ, et al (1996) Proteins encoded by open reading frames 3 and 4 of the genome of Lelystad virus (Arteriviridae) are structural proteins of the virion. J Virol 70: 4767–4772. 8676504 10.1128/jvi.70.7.4767-4772.1996 PMC190414

[23] Rodriguez MJ, Sarraseca J, Garcia J, Sanz A, Plana-Duran J, et al (1997) Epitope mapping of the nucleocapsid protein of European and North American isolates of porcine reproductive and respiratory syndrome virus. J Gen Virol 78: 2269–2278. 9292014 10.1099/0022-1317-78-9-2269

[24] Plana Duran J, Climent I, Sarraseca J, Urniza A, Cortes E, et al (1997b) Baculovirus expression of proteins of porcine reproductive and respiratory syndrome virus strain Olot/91. Involvement of ORF3 and ORF5 proteins in protection. Virus Genes 14: 19–29. 9208452 10.1023/a:1007931322271

[25] Cancel-Tirado SM, Evans RB, Yoon KJ (2004) Monoclonal antibody analysis of porcine reproductive and respiratory syndrome virus epitopes associated with antibody-dependent enhancement and neutralization of virus infection. Vet Immunol Immunop 102: 249–262. 10.1016/j.vetimm.2004.09.017 PMC7173136 15507309

[26] Zhou YJ, Hao XF, Tian ZJ, Tong GZ, Yoo D, et al (2008) Highly virulent porcine reproductive and respiratory syndrome virus emerged in China. Transbound Emerg Dis 55: 152–164. 18405338 10.1111/j.1865-1682.2008.01020.x

[27] Leng CL, An TQ, Chen JZ, Gong DQ, Peng JM, et al (2012) Highly pathogenic porcine reproductive and respiratory syndrome virus GP5 B antigenic region is not a neutralizing antigenic region. Vet Microbiol 159 (3): 273–281. 10.1016/j.vetmic.2012.06.018 22771210

[28] Larochelle R, Magar R (1997) Evaluation of the presence of porcine reproductive and respiratory syndrome virus in packaged pig meat using virus isolation and polymerase chain reaction (PCR) method. Vet Microbiol 58: 1–8. 9451456 10.1016/s0378-1135(97)00150-8

[29] Plagemann PGW, Rowland RRR, Faaberg KS (2002) The primary neutralization epitope of porcine reproductive and respiratory syndrome virus strain VR-2332 is located in the middle of the GP5 ectodomain. Arch Virol 147: 2327–2347. 12491101 10.1007/s00705-002-0887-2

[30] Katz JB, Shafer AL, Eernisse KA, Landgraf JG, Nelson EA (1995) Antigenic differences between European and American isolates of porcine reproductive and respiratory syndrome virus (PRRSV) are encoded by the carboxyterminal portion of viral open reading frame 3. Vet Microbiol 44: 65–76. 7667907 10.1016/0378-1135(94)00113-B PMC7117291

[31] Gonin P, Mardassi H, Gagnon CA, Massie B, Dea S (1998) A nonstructural and antigenic glycoprotein is encoded by ORF3 of the IAF-Klop strain of porcine reproductive and respiratory syndrome virus. Arch Virol 143: 1927–1940. 9856081 10.1007/s007050050430 PMC7086821

[32] Hedges JF, Balasuriya UB, MacLachlan NJ (1999) The open reading frame 3 of equine arteritis virus encodes an immunogenic glycosylated, integral membrane protein. Virology 264: 92–98. 10544133 10.1006/viro.1999.9982

[33] Oleksiewicz MB, Botner A, Normann P (2002) Porcine B-cells recognize epitopes that are conserved between the structural proteins of American- and European-type porcine reproductive and respiratory syndrome virus. J Gen Virol 83: 1407–1418. 12029156 10.1099/0022-1317-83-6-1407

[34] Zhou YJ, An TQ, He YX, Liu JX, Qiu HJ, et al (2006) Antigenic structure analysis of glycosylated protein 3 of porcine reproductive and respiratory syndrome virus. Virus Res 118(1): 98–104. 16384621 10.1016/j.virusres.2005.11.019

[35] Li Y, Jia Y, Wen K, Liu H, Gao MC, et al (2013) Mapping B-cell linear epitopes of NS3 protein of bovine viral diarrhea virus. Vet Immunol Immunopathol 151: 331–336. 23276751 10.1016/j.vetimm.2012.11.011

